# Rationality and suicide, cultural context and mental illness – tenuous limits: about a clinical case

**DOI:** 10.1192/j.eurpsy.2022.1401

**Published:** 2022-09-01

**Authors:** C. Freitas, M. Felizardo

**Affiliations:** Centro Hospitalar de Trás-os-Montes e Alto Douro, Psiquiatria, Vila Real, Portugal

**Keywords:** Rational suicide, Cultural, Suicide, mental illness

## Abstract

**Introduction:**

Suicide results from a complex interaction between biological, genetic, psychological, sociological, cultural and environmental factors. The frequency of suicide among psychiatric pathologies is quite variable, with depression accounting for 45% to 70% of suicides. The association of suicide with the existence of mental illness is not consensual, with reports of rational suicides in 2% to 9% of suicide cases. It is unquestionable that the awareness of the lived experience limits the person’s condition to what it is.

**Objectives:**

To describe a clinical case on the subject and discuss the influence of cultural context in suicide.

**Methods:**

The authors describe a case of a patient hospitalized in Psychiatry, after a suicide attempt and a consummated suicide by his wife.

**Results:**

The patient and his wife lived their entire lives as hermits. Although no acute psychopathology was found in the patient to justify the act, such as psychotic or depressive symptoms, dysfunctional personality traits were found, which translated into an attitude of superiority, requirement of subservience, hostility when contradicted and breaches of basic rules.

**Conclusions:**

Taking into account what has been described, the authors discuss the influence of personality on the patient’s life choices and on the decision that led to the suicide attempt, as well as the suicide of his wife. A reflection is made on whether suicide can be completely independent of mental illness or whether, even in cases where rationality seems to be the causal factor, personality dysfunctionality and a profound influence of the cultural context, are present or not.

**Disclosure:**

No significant relationships.

